# The Actin-Bundling Protein L-Plastin: A Critical Regulator of Immune Cell Function

**DOI:** 10.1155/2012/935173

**Published:** 2011-12-13

**Authors:** Sharon Celeste Morley

**Affiliations:** Division of Infectious Diseases, Department of Pediatrics, Washington University School of Medicine, St. Louis, MO 63110, USA

## Abstract

L-plastin is a leukocyte-specific protein that cross-links actin filaments into tight bundles, increasing the stability of actin-based structures such as podosomes and lamellipodia. While first identified as an abundant cytoplasmic protein in hematopoietically derived cells over 25 years ago, the requirement for L-plastin in multiple functions critical for immunity, such as antigen receptor signaling, adhesion, and motility, has only recently become clear. L-plastin has been identified as an important component in cellular processes critical for neutrophil, macrophage, osteoclast, eosinophil, and T- and B-lymphocyte biology. Following a brief description of the structure and function of L-plastin, the regulation of immune cell functions by L-plastin will be reviewed in detail.

## 1. Introduction

The actin cytoskeleton enables numerous cellular processes required for the mammalian immune response. Actin is rapidly polymerized in response to T-cell receptor signaling, and F-actin provides stability for the contact site between antigen-presenting cells and T cells, termed the immunological synapse [1–4]. Actin cytoskeletal elements are also recruited during chemotactic and adhesive responses, processes critical to normal leukocyte trafficking and motility [5–10]. Phagocytosis and intracellular killing of pathogens also relies upon actin cytoskeletal elements [11, 12]. While many actin-binding proteins regulate the recruitment and stabilization of the actin cytoskeleton, recent studies have implicated the actin-bundling protein L-plastin (LPL) as a critical regulator of actin dynamics in cells of both the adaptive and innate immune systems.

## 2. LPL Expression, Structure, and Function

**Figure 1:**
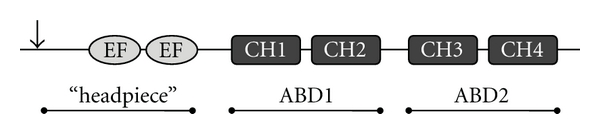
Schematic of the structure of LPL. The N-terminal “headpiece” contains at least one serine phosphorylation site (arrow; serine residue 5) and EF hand loops (labeled “EF”) that are thought to participate in the calcium regulation of LPL. The C-terminal portion contains two tandem ABDs, each of which consists of two calponin-homology (CH) domains, which are numbered.

Plastins, or fimbrins, are actin-bundling proteins critical to actin regulation in eukaryotes. Human fimbrin can complement yeast SAC6 in endocytosis, suggesting a high degree of conservation [13]. Three isoforms, L-, I-, and T-plastin, comprise the vertebrate plastins. LPL was initially found in transformed human fibroblasts, though it was later recognized that normal expression of LPL is restricted to cells of the hematopoietic lineage [14–18]. LPL, also called lymphocyte cytosolic protein 1 (LCP1), has been described as one of the 15 most abundant proteins in human monocytes and T cells [14]. The human isoform I-plastin is expressed in mammalian small intestine, colon, and kidney [19, 20]. T-plastin has the broadest tissue distribution and has been found in most cells from solid tissues with replicative potential, such as fibroblasts and epithelial cells [16, 18]. All three plastins contain two N-terminal EF-hands, homologous to calmodulin-calcium-binding domains, followed by two actin-binding domains (ABDs). Unlike I- and T-plastin, LPL additionally contains N-terminal sites of serine phophorylation (Figure 1).

Plastins bind F-actin through ABDs that each consists of two calponin-homology domains, placing plastins in the *α*-actinin family. Other *α*-actinin family members include *α*-actinin, filamin, spectrin, dystrophin, and actin-binding protein 120 [16, 21]. Plastins are unique among these family members in that they contain two tandem ABDs on the same polypeptide. These tandem ABDs are thought to fold into a compact, horseshoe-like structure that can simultaneously bind two actin filaments, thus cross-linking the filaments into tight bundles [22]. An atomic model of actin filament bundling by T-plastin was generated through electron microscopy of 2D actin arrays polymerized in the presence or absence of T-plastin on lipid bilayers [23, 24]. Bundled actin arrays were unipolar, with about 120 Å between filaments, and bundling may generate hexagonal lattices [24]. Incorporation of T-plastin into actin bundles occurred primarily during actin polymerization; cross-linking of filaments was irregular when preformed actin filaments were incubated with T-plastin. T-plastin-bundled filaments could bend at sites of T-plastin cross-links, likely through changes in the twist of the bound actin filament [24].

**Figure 2:**
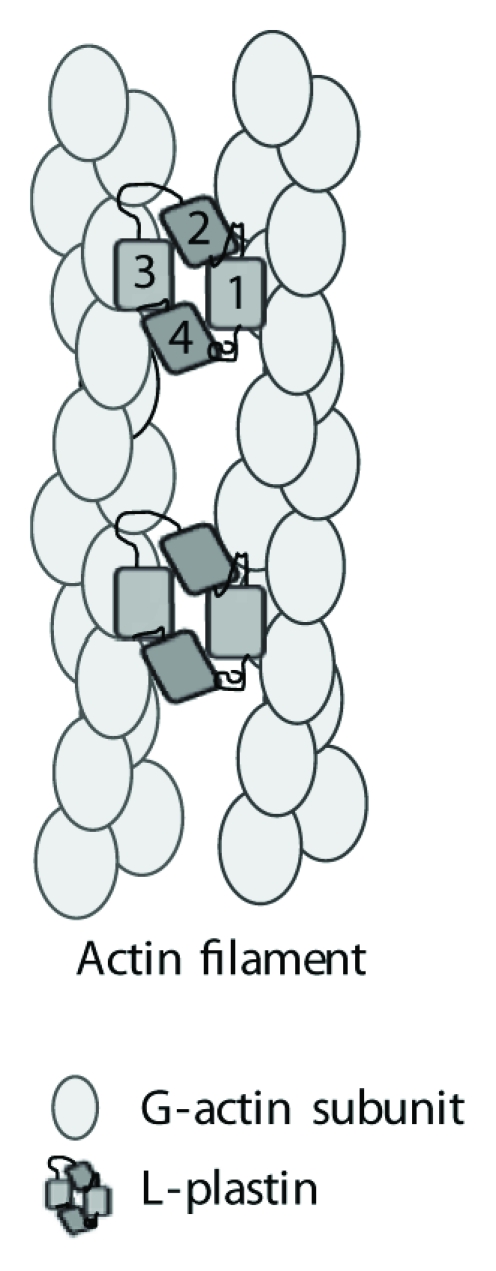
LPL folds into a compact structure that bundles actin filaments. Numbers indicate each CH domain. Based on available experimental data, it has been proposed that ABD2 binds to a polymerizing actin filament such that the nucleotide-binding cleft adopts a more “closed” position, thus stabilizing the filament. Binding of ABD1 to an adjacent filament cross-links F-actin into tight bundles.

While LPL is thought to bundle actin in a manner similar to the homologous T-plastin (Figure 2), direct confirmation through experimental demonstration has been elusive. Imaging of actin filaments has been limited by the intrinsic disorder of F-actin, as filaments contain variable twist and tilt of actin subunits. This limitation was overcome by using high-resolution cryoelectron microscopy to image the binding of LPL to F-actin [25]. These images of LPL-decorated F-actin revealed that binding of the ABD2 of LPL reduced the variability in actin twist and resulted in a more “closed” conformation of the nucleotide-binding cleft in actin subunit. As an “open” conformation of the nucleotide-binding cleft is correlated with depolymerization and actin filament instability, “closure” of the cleft by LPL offers a molecular understanding of prior results indicating that LPL binding stabilized the polymerized actin filament [26]. The interaction of the ABD1 of LPL with F-actin was too variable to be described using the same technique of cryo-electron microscopy, although a prior report indicated that the binding of ABD1 of LPL likely resembles the binding of *α*-actinin [25, 26]. It is unclear whether the ABD1 or the ABD2 of LPL first binds to F-actin [25, 26]. While the exact molecular mechanism of LPL bundling to F-actin remains to be experimentally delineated, the binding sites of ABD1 and ABD2 have been described [26], and it seems likely that LPL functions similarly to T-plastin in cross-linking filaments [24].

No molecular function beyond actin bundling has been described for LPL. However, as outlined below, peptides derived from the N-terminus of LPL lacking ABD domains can promote changes in cell functions, such as integrin avidity and cell adhesion. These results suggest that LPL may play a role as an adaptor or a scaffolding protein in signaling in addition to its actin-bundling activity, roles that require further experimental elucidation. Also, as will be discussed below, the details of mechanisms by which actin bundling may promote or restrain immune cell functions have not yet been fully described.

## 3. Regulation of LPL

Regulation of the bundling of activity of LPL has been demonstrated to occur through both calcium binding and serine phosphorylation. LPL is phosphorylated in multiple cell types primarily at serine residue 5 following a variety of stimuli, including IL-1, IL-2, lipopolysaccharide, fMet-Leu-Phe, Fc*γ*R ligation, and PMA [27–31]. To study the effect of serine phosphorylation on the binding of LPL to F-actin, the serine 5 residue was mutated to either a nonphosphorylatable alanine residue (S5A mutant) or to a glutamic acid residue (S5E mutant), that mimicked constitutive phosphorylation [32]. Wild-type and mutant LPL were expressed in Vero cells, which do not express endogenous LPL. Phosphorylation of LPL was found to enhance the targeting of LPL to F-actin rich structures and regions of rapid actin assembly, including membrane ruffles and microspikes. Phosphorylation of LPL increased its binding to F-actin, as the S5E mutant LPL had a much higher actin-bundling activity than did wild-type LPL [32]. Analysis of fluorescence recovery after photobleaching (FRAP) of GFP-tagged wild-type and S5A mutant LPL in Vero cells revealed that phosphorylation of LPL increased its localization to focal adhesion sites and reduced its lateral mobility, again suggesting an increase in binding of phosphorylated LPL to F-actin [33]. Phosphorylated LPL also reduced the turnover of actin filaments in focal adhesions to a greater extent than non-phosphorylatable LPL, although nonphosphorylatable LPL also stabilized F-actin [33]. Thus, when ectopically expressed in Vero cells, serine phosphorylation may directly regulate the bundling function of LPL.

LPL may differ from I- and T-plastin in that calcium binding negatively regulates LPL-bundling activity [34]. In an *in vitro* assay, it was determined that calcium concentrations of greater than 10^−6 ^M reduced the binding of LPL to F-actin, while LPL binding to F-actin was intact at concentrations of less than 10^−7 ^M calcium [34]. Calcium binding may induce conformational changes in LPL, thus altering its actin-binding ability [22]. Phosphoinositides have also been reported to reduce the interaction between LPL and actin filaments [26].

LPL may also be regulated through direct binding by other proteins. For instance, binding of the protein-ionized calcium binding adaptor molecule 1 (Iba1) to LPL in macrophages enhanced the bundling capability of LPL, independently of the intrinsic bundling capability of Iba1 [35]. Iba1 is a macrophage-specific, actin-bundling protein that localizes to membrane ruffles and participates in phagocytosis [35]. Binding of LPL to cortactin has been described in Vero cells in which LPL has been ectopically expressed [33]. The macrophage-specific protein grancalcin may bind LPL directly [36], and vimentin and LPL are complexed in adherent macrophages [37]. Finally, calmodulin binding may affect the function of LPL in stimulated T cells [38]. Whether the binding of cortactin, grancalcin, or calmodulin modulates the bundling capacity of LPL has not been determined.

Despite much work on the various mechanisms by which LPL may be regulated, an integrated description of how these mechanisms interact during a specific cell process in a specific cell type, such as macrophage adhesion, has not emerged. Such an integrated description is challenging, because the regulation of LPL likely varies from cell type to cell type and from stimulus to stimulus. In fact, different receptors for the same stimulus can regulate LPL via different signaling pathways [39]. However, given the abundance of LPL in hematopoietic cells, and its recruitment into a wide variety of processes critical to immune function, a more thorough exploration of the regulation of LPL is warranted.

## 4. LPL Regulates Integrin Function in Neutrophils

The most extensive characterization of a role for LPL in hematopoietic cells has been performed in neutrophils. Critical to the immediate, innate host defense, neutrophils rely on complex interactions between Fc receptors, chemoattractant receptors, and integrins to guide neutrophil maturation, trafficking, and degranulation [40]. Regulation of integrin avidity through inside-out signaling and integrin-mediated outside-in signaling is crucial to effective neutrophil function [41–43]. The predominance of evidence indicates that LPL participates in both inside-out and outside-in signaling [29, 44], although LPL may also function downstream of Fc*γ*R in some systems.

LPL was initially recognized in neutrophils as a target of phosphorylation following stimulation with IL-8 or the chemoattractant peptide fMet-Leu-Phe [45, 46]. LPL was independently found to be a downstream mediator of Fc*γ*R signaling when LPL was identified as a target of bromophenacyl bromide (BPB) [47]. BPB inhibited Fc*γ*R-mediated calcium flux and diminished neutrophil degranulation, adherence, and phagocytosis. BPB-mediated inhibition of Fc*γ*R signaling correlated with the binding of BPB to LPL. Intriguingly, BPB did not prevent the Fc*γ*R-stimulation binding of LPL to F-actin, suggesting that LPL may have a function beyond that of actin bundling in Fc*γ*R signaling in neutrophils [47]. Further work revealed that Fc*γ*RII activation through bead- or plate-bound immune complexes induced phosphorylation of the N-terminal headpiece of LPL, while stimulation with soluble immune complexes did not [48]. Immune complexes consisted of bovine serum albumin (BSA) bound with varying concentrations of anti-BSA antibodies. Phosphorylation of LPL under conditions in which cells had to reorganize to engage with a fixed ligand but not in response to simple receptor triggering by a soluble ligand suggested that LPL may be involved in the cellular shape change induced by binding to fixed ligands [48]. Furthermore, plate-bound immune complexes also induced the movement of LPL to podosomes. However, LPL phosphorylation occurred independently of the translocation to podosomes, calcium flux, and actin polymerization. Blockade of the integrin *α*M*β*2 (also called CR3, Mac-1, and CD11b/CD18) did not affect LPL phosphorylation, but did reduce the translocation of LPL to the podosomes on immune complex coated surfaces [48]. Thus, it was not clear in early work whether the phosphorylation of LPL promoted association with F-actin in neutrophils stimulated through plate-bound Fc*γ*R, and the precise regulation and function of LPL in neutrophils adhering to surface-bound ligands remain to be fully described.

A mechanistic link between phosphorylation of LPL and activation of the integrin *α*M*β*2 in neutrophils was revealed in human neutrophils treated with a cell-permeable synthetic peptide derived from the N-terminal headpiece of LPL [29]. Signals that generate changes in integrin adhesion also triggered LPL phosphorylation; LPL phosphorylation in neutrophils could be induced by both PI3K-dependent Fc*γ*R signaling and by PI3K-independent f-Met-Leu-Phe and PMA stimulation. The serine 5 residue in the N-terminus of LPL was identified as the primary target of phosphorylation in these signaling pathways. A synthetic peptide derived from residues 2–19 of the N-terminus of LPL was fused at the carboxy terminus to the HIV *tat* protein, which enabled spontaneous translocation of the LPL peptide across the cell membrane. Treatment of neutrophils with the wild-type sequence of LPL was sufficient to induce adhesion of neutrophils to surfaces coated with fetal calf serum, a process mediated through the activation of the integrin *α*M*β*2. Treatment with a constitutively phosphorylated synthetic LPL peptide also induced adhesion, while mutation of the serine residue at position 5 to alanine to prevent phosphorylation abrogated the induction of neutrophil adhesion. Induction of adhesion by wild-type LPL*tat* but not by constitutively phosphorylated LPL*tat* could be inhibited by blocking PI3K and PKC, indicating that phosphorylation of LPL through these signaling pathways was likely necessary for the ability of LPL to induce activation of *α*M*β*2 and thus neutrophil adhesion [29]. Additionally, blockade of LPL phosphorylation by treatment with the PKA-inhibitor H89 correlated with inhibition of adhesion to immune complex coated surfaces and likely inhibited activation of *α*M*β*2 [49].

Induction of integrin activation was confirmed and extended through the examination of activation of *α*v*β*3 in the human erythroleukemic cell line K562, which enabled analysis of the function of LPL in a well-established system of activable integrin adhesion [50]. Similar to the observation that a synthetic LPL peptide could upregulate the adhesion of *α*M*β*2 in neutrophils [29], treatment of K562 cells expressing *α*v*β*3 with the LPL*tat* peptide promoted integrin activation and cellular adhesion to surfaces coated with the integrin ligand vitronectin [50]. LPL*tat* activation of *α*v*β*3 was independent of the tyrosine phosphorylation of the cytoplasmic tail of the integrin *β* chain, indicating that LPL activates integrins in a mechanism different from that of PMA stimulation. LPL*tat* treatment of cells triggered a conformation change in the integrin, revealing ligand-induced binding sites on the integrin that increased its avidity for its ligand. Changes in integrin avidity required actin depolymerization [50]. These results confirmed that the action of LPL was not restricted to one specific integrin, and further elucidated the mechanism by which LPL can activate integrins in neutrophils and K562 cells. Whether LPL bound directly to integrins *α*M*β*2 and *α*v*β*3 was not examined in these studies. However, LPL has recently been reported to exist in complexes with either *β*1 or *β*2 integrins in breast and prostate cancer cells, consistent with the proposal that LPL can directly regulate integrin activity [21].

## 5. Regulation of LPL Phosphorylation in Neutrophils

Phosphorylation of LPL has also been linked to generation of NADPH-oxidative burst in neutrophils following stimulation with PMA. Both nonphosphorylated and hyperphosphorylation of LPL correlated with inhibition of PMA-generated NADPH oxidative burst. Optimally phosphorylated LPL correlated with induction of NADPH oxidative burst [51]. These results suggested turnover of LPL phosphorylation may be as critical as phosphorylation itself. Whether or not that turnover of LPL phosphorylation functions in the generation of the oxidative burst in response to other extracellular signals has not been investigated in this manner.

The kinases that phosphorylate LPL in neutrophils vary with cell stimulus (Table 1). Immune complex binding triggers phosphorylation of LPL at the serine 5 residue through PKA [49]. However, the inhibitor of PKA, H89, did not inhibit LPL phosphoryaltion in response to PMA or fMLP stimulation [49]. Characterization of LPL phosphorylation in response to fMLP revealed multiple signaling pathways that converge at LPL phosphorylation. Inhibitors of PI3K, PLD, and PKC could all reduce LPL phosphorylation in response to fMLP stimulation, and the recruitment of the different kinases depended upon whether signaling occurred through the high-affinity or low-affinity receptor of fMLP [39]. LPL was independently found to be a substrate of phosphorylation following ligation of the low-affinity receptor for fMLP, formyl peptide receptor-like 1 (FPR-L1) [30]. Intriguingly, multiple species of LPL—a 67 kDa, 65 kDa, 62 kDa, and 48 kDa—were isolated from fMLP-stimulated neutrophils. The signficance of these different molecular weight forms of LPL is completely unknown [30].

Given the increase in integrin activation and adhesion triggered by the LPL*tat* peptide, it was somewhat of a surprise to find that neutrophils from the LPL^−/−^ mouse were not defective in integrin-mediated adhesion, nor was chemotaxis of LPL^−/−^ neutrophils diminished [44]. However, LPL^−/−^ neutrophils were defective in integrin signaling to the kinase Syk. Deficient activation of Syk following integrin ligation led to a defective respiratory burst, resulting in an inability to kill *Staphylococcus aureus*, and LPL^−/−^ mice were more susceptible to staphylococcal skin abscesses. Signaling through both Fc*γ*R and PMA to the respiratory burst in LPL^−/−^ neutrophils was intact, indicating that integrin signaling was specifically dependent upon LPL [44]. These results, combined with those described above, suggest that LPL may be sufficient to promote changes in integrin adhesion, but is not always necessary. However, LPL is required for some aspects of integrin outside-in signaling.

## 6. LPL Promotes the Stabilization of the T-Cell Immune Synapse

In addition to its role in neutrophil biology, LPL has also been recognized as a substrate of phosphorylation during T-cell receptor signaling to T-cell activation as early as 1994 [52]. Phosphorylation or redistribution of LPL has been used as a marker of T-cell activation and/or costimulation [53, 54]. However, a functional requirement for LPL during T-cell activation has only recently been described [55, 56].

T-cell activation is dependent upon the creation of a specialized contact site between the antigen-presenting cell (APC) and the responding T-cell [57, 58]. Described as both the supramolecular activation complex (SMAC) and the immunological synapse (IS), the creation of this contact site has been extensively reviewed [2, 3, 59]. A role for LPL in the formation of the IS was suggested by the findings that LPL accumulates at the synapse [55]. Co-stimulation through CD2 or CD28 induced phosphorylation of LPL at serine 5 [55]. While accumulation of LPL at the IS was independent of its phosphorylation status, overexpression of a mutant of LPL that could not be phosphorylated reduced the export of the T-cell activation markers CD25 and CD69 [55]. An additional study from the same group further characterized the role of LPL in the formation of the mature IS [38]. They found that during SMAC formation, LPL primarily colocalized with F-actin in the peripheral and/or distal SMAC. As in the previous study, mutation of the serine phosphorylation site at residue 5 had no impact on the ability of LPL to localize to the IS. However, deletion of an ABD or deletion of the calmodulin-binding domain did prevent LPL accumulation at the synapse. Knockdown of LPL through siRNA transfection resulted in diminished actin polymerization during synapse formation, reduced recruitment of talin and LFA-1 to the synapse, and an overall reduction in the size of the synapse. Defective IS maintenance correlated with a reduction in the proliferation of peripheral blood T cells in which LPL had been knocked down [38]. Recently, phosphorylation of LPL has been identified as a target of dexamethasone, offering a novel mechanism for the immunosuppressive effects of this commonly used steroid [61].

A requirement for LPL in the formation of the immune synapse was independently confirmed through the analysis of murine T cells genetically deficient for LPL [56]. LPL^−/−^ murine T cells were defective in proliferation and cytokine production when stimulated with plate-bound anti-CD3 or with peptide-pulsed APCs, but not with soluble anti-CD3. Interestingly, proximal TCR signaling events, such as calcium flux, tyrosine phosphorylation of the adaptor protein LAT, and ERK activation and were intact in stimulated LPL^−/−^ T-cells. However, spreading of LPL^−/−^ T cells on anti-CD3 coated surfaces, formation of LPL^−/−^ T-cell: APC conjugates, and generation of the IS in LPL^−/−^ T cells were impaired. These data are all consistent with a model in which LPL is required for later stages of IS formation, stabilization, or maintenance, but is dispensable for early TCR signaling events. A requirement for LPL in later stages of signaling would also be consistent with its identified functions in stabilizing F-actin structures. Defective formation or maintenance of the IS likely contributed to the downstream defects in proliferation and cytokine production of LPL^−/−^ T cells [56]. It is not yet clear whether the requirement for LPL in stabilization of the IS depends upon its actin-bundling activity, effects upon integrin binding or adhesion, binding to calmodulin, or some combination of these potential effector functions. Furthermore, the kinases that phosphorylate LPL and the potential regulation of LPL by calcium, as is suggested by the binding of calmodulin to LPL [38], have not yet been fully elucidated in TCR-stimulated T cells. Furthermore, while B-cell activation also requires actin rearrangement, a role for LPL in B-cell receptor signaling has not yet been fully investigated [62].

## 7. LPL Is Required for Normal T- and B-Cell Motility

Lymphocyte trafficking is dependent upon an array of integrins and chemoattractant receptors that promote changes in cell polarity, adhesion, and motility [8, 63–66]. LPL has also been recognized as a critical regulator of T-cell motility, as well as T-cell activation. Like its role in TCR signaling, LPL appears to function as a downstream effector of motility, rather than as a participant in proximal chemokine receptor signaling. LPL was identified along with coronin 1A as one of several actin-binding proteins that move into and then out of chemokine-receptor-associated lipid rafts following chemokine signaling. Knockdown of LPL in Jurkat T cells reduced motility towards CCL20 without inhibiting chemokine-stimulated calcium flux [67]. T cells from LPL^−/−^ mice were also found to have diminished chemoattractant-mediated motility, as assessed by two-photon microscopy in explanted lymph nodes as well as in transwell assays [68]. The reduction in motility occurred in chemokine-stimulated LPL^−/−^ T cells despite normal activation of the small GTPase Rac, induction of a burst of actin polymerization, and upregulation of cellular adhesiveness thought to be due to integrin activation. Motility reduction correlated with diminished polarization of LPL^−/−^ T cells, which failed to generate clearly delineated uropods and lamellipods seen in chemokine-stimulated WT T cells. Phenotypically, the reduced motility of LPL^−/−^ T cells resulted in diminished thymic egress in LPL^−/−^ mice [68]. While inhibition of immune cell motility by an inverse agonist of the cannabinoid CB2 receptor correlated with diminished phosphorylation of LPL [69], a requirement for LPL phosphorylation during T-cell chemotaxis has not yet been formally demonstrated, and whether calmodulin-binding regulates the function or localization of LPL during chemotaxis has not been examined.

B cells also require LPL for chemotaxis-mediated motility [70]. LPL^−/−^ mice were found to be deficient for a specialized population of B cells, called marginal zone B cells. Development of MZ B cells is exquisitely sensitive to chemotactic and adhesive signaling, and LPL^−/−^ B cells were found to exhibit reduced motility towards the chemoattractants CXCL12, CXCL13, and sphingosine-1-phosphate. Interestingly, the integrin-mediated increase in cellular motility required LPL, while the chemokine-mediated increase in integrin adhesiveness did not. As seen before, some downstream elements of chemoattractant receptor signaling did not require LPL, as activation of ERK and p38 were intact in CXCL12-stimulated LPL^−/−^ B cells. However, total protein levels and phosphorylation of the integrin-associated kinase Pyk2 were diminished in LPL^−/−^ B cells, providing a possible molecular explanation for the requirement for LPL in lymphocyte motility [70].

## 8. LPL Is an Early Participant in Sealing Ring Formation of Osteoclasts

Osteoclasts are highly specialized, hematopoietically derived cells that maintain bone mass and remodel bone structure through the tightly regulated process of bone resorption [71]. LPL has been localized to the podosomes of monocyte-derived osteoclasts [72], and a role for LPL, along with the actin-binding protein cortactin, has been demonstrated in osteoclast sealing ring formation [73]. Osteoclasts undergo dramatic, actin-based morphologic changes during bone resorption. Osteoclasts initially generate actin aggregates, which are thought to provide traction for membrane extensions. These actin aggregates then mature and form a sealing ring. The expression of LPL was found to decrease during the maturation of the sealing ring, while the expression of cortactin increases during the same time period. LPL was found to localize with the actin aggregates during the early process of bone resorption, but not with the mature sealing ring. While an absolute requirement for LPL in the formation of the sealing ring has not yet been formally demonstrated, these results suggest that LPL plays a critical function during osteoclast adhesion [73].

## 9. LPL Localizes to the Podosomes of Macrophages during Migration

LPL was identified as one of the major constituents of cytoplasmic gels isolated from alveolar macrophages, along with actin, filamin, and *α*-actinin [17]. LPL was estimated to account for about 1.8% of total cytoplasmic protein in macrophages, existing in an approximate 1 : 7-8 molar ratio with actin. The authors proposed that calcium regulation of LPL may alter microfilament organization in macrophages, as the presence of 20 *μ*M calcium reduced the affinity of LPL for actin by threefold. LPL was also identified as a heavily phosphorylated protein in macrophages stimulated with LPS [74]. As seen in other cell types, the phosphorylation occurs at serine residue 5 of the amino terminus, though the kinase responsible for LPL phosphorylation in LPS-stimulated macrophages remains undefined [31, 74]. A small amount of constitutively phosphorylated LPL has been reported to associate with podosomes in macrophages [75].

The abundance of LPL in macrophage cytoplasm has enabled the imaging of macrophage movement through fluorescently tagged LPL [76]. Fluorescently tagged LPL has also been used in conjunction with fluorescently labeled actin to analyze podosome assembly and disassembly in macrophage lamellipodia during migration [77]. Podosomes are similar to focal adhesions and serve as sites of actin assembly and adhesion during forward movement and are enriched for integrins, vinculin, and talin. Podosomes are relatively short lived, assembling and disassembling in a few minutes. Dynamic changes in F-actin and LPL were tightly associated both geographically and temporally, suggesting close coupling between the regulation of the two proteins [77]. As LPL is incorporated during actin polymerization [24], this close coupling in time and space is not surprising. While LPL is certainly an abundant component of podosomes, a requirement for actin bundling in normal podosome assembly and function has not yet been reported.

LPL has also been reported to bind to macrophage-specific proteins that may be critical to macrophage function. For instance, LPL was found to bind Iba1 in a two-hybrid screen [35]. The expression of Iba1, an EF-hand protein, is upregulated in activated microglia and has been reported to be critical for macrophage membrane ruffling and phagocytosis. Iba1 and LPL co-localize to phagocytic cups. Binding of Iba1 to LPL enhanced LPL bundling activity, independent of the intrinsic bundling ability of Iba1 [35]. LPL was also found to be a possible binding partner of grancalcin, a member of the penta-EF-hand family [78]. Grancalcin is expressed primarily in phagocytic cells, such as neutrophils and macrophages [36]. Grancalcin colocalized with F-actin in membrane spikes of stimulated macrophages [36]. Unlike LPL^−/−^ neutrophils, grancalcin-deficient neutrophils were able to kill *Staphylococcus aureus in vitro*, and grancalcin was not required for other immune functions, such as macrophage recruitment to sites of inflammation or resistance to fungal infections [79]. The functional association between LPL and grancalcin thus remains undefined.

## 10. LPL Mediates GM-CSF Sensitization of Eosinophils

A function of LPL in receptor signaling events beyond its capacity as an actin-bundling protein has been suggested by a recent report that LPL may regulate GM-CSF-mediated sensitization of eosinophils [60]. GM-CSF stimulation promoted the phosphorylation of LPL and the association of LPL with protein kinase C*β*II and two subunits of the GM-CSF receptor. Treatment with GM-CSF resulted in increased expression of the integrin *α*M*β*2, increased sensitivity to eotaxin in a chemotaxis assay, prolonged survival of eosinophils, and primed eosinophils for degranulation. Inhibition of PKC*β*II resulted in the reduction of LPL phosphorylation and the loss of these GM-CSF-stimulated effects. Most importantly, internalization of a constitutively phosphorylated LPL peptide (residues 2–19) by eosinophils resulted in increased *α*M*β*2 integrin expression and sensitization to chemotaxis, suggesting a mechanistic link between phosphorylation of LPL and GM-CSF-induced sensitization to chemotaxis [60]. The induction of increased *α*M*β*2 integrin expression by the N-terminal portion of LPL, which does not contain ABDs, suggests that LPL may have intrinsic signaling capabilities that do not require actin-bundling functions.

## 11. LPL May Promote the Invasive Potential of Transformed Cells

LPL is ectopically expressed in many tumor cells, and its function in the potential promotion of invasive potential of transformed cells has been extensively reviewed [80]. In brief, a survey of 59 human tumor cell lines revealed ectopic expression of LPL in 66% of epithelial-derived carcinomas and 53% of mesenchymal tumors [18]. Expression of LPL has been correlated with higher stages of colorectal cancer [81], and ectopic expression of LPL in a colon cancer cell line promoted proliferation and invasion [82]. While total expression of LPL did not correlate with staging of breast cancer [83], the phosphorylation of LPL enabled breast cancer cell line resistance to TNF-*α* [84]. The invasive potential of human melanoma cells was enhanced by the phosphorylation of ectopically expressed LPL [85]. Blockade of LPL by the overexpression of an alpaca-derived nanobody reduces invasion of prostate carcinoma cells in a matrigel invasion assay [86]. Finally, a nonphosphorylatable mutant of LPL was unable to promote collagen gel invasion of HEK293T cells [32]. Combined, these results suggest that LPL may function in the formation of actin-based structures that facilitate the metastatic potential of transformed cells [80].

## 12. Toward an Integrated Model of LPL

Despite 25 years of research, an integrated understanding of the function of LPL in hematopoietic cells remains elusive. A clear description of the role of LPL has been frustrated by the inherent technical difficulties of analyzing the rapid, transient, and subcellular events of receptor signaling, adhesion, and motility that are dependent upon the highly dynamic actin cytoskeleton. Advances in imaging technology will address these difficulties. Additionally, the requirement for LPL may vary with cell type and cell stimulus, as LPL was required for chemoattractant-mediated motility of lymphocytes [68, 70], but not migration of neutrophils [44]. It may be that the differential requirement for LPL in lymphocytes and neutrophils results from the utilization of different modes of motility [10, 87]. It is also possible that the differential requirement for LPL in distinct cell types results from disparate expression patterns of LPL-binding proteins, such as grancalcin [36]. Furthermore, it has not yet been established whether LPL regulates receptor signaling, adhesion, and motility solely through its actin-bundling function or if LPL can also act as an adaptor protein in signaling cascades.

A function for LPL beyond actin-bundling has been recently suggested by the observation that LPL binds directly to the cytoplasmic portion of integrins *β*1 and *β*2 though the ABDs [21]. This interaction between LPL and integrins *β*1 and *β*2 may be regulated by *μ*-calpain cleavage [21]. LPL was also identified in a screen for proteins upregulated in a leukemic cell line that constitutively maintains integrins in an activated state [88]. A critical role for LPL in integrin signaling would reconcile a number of otherwise apparently disparate observations. For instance, stabilization of the IS in T cells, the adhesion-mediated triggering of the NADPH oxidative burst in neutrophils, and the stabilization of F-actin in the podosomes of macrophages are all integrin-mediated events [38, 44, 56, 77]. The requirement for LPL in signaling has often been revealed when suspension cells, such as neutrophils or T cells, were stimulated with surface-bound, but not soluble, ligand, consistent with the proposal that LPL functions primarily in transmitting adhesion-generated signals [48, 56]. The demonstration of defective polarization of chemokine-stimulated LPL^−/−^ T cells was performed on a surface coated with integrin ligand [68]. Whether LPL regulates “inside-out” integrin signaling may be cell type dependent. LPL upregulated integrin avidity in neutrophils [29], but upregulation of integrin avidity following chemokine stimulation was not LPL-dependent in lymphocytes [68, 70]. Integrin outside-in signaling required LPL in both lymphocytes and neutrophils [44, 68, 70]. Whether LPL links integrins directly to the actin cytoskeleton or serves as an adaptor protein in a larger molecular complex remains unclear.

Mutations in cytoskeletal proteins, such as Wiskott-Aldrich Syndrome protein, coronin, DOCK8 and Rac2, result in human immunodeficiency [89–92]. Whether mutations in or allelic forms of LPL contribute to human immunodeficiency is an open question. One of the first reports of LPL noted an alternatively expressed form in one of nine patients examined [14]. It does not appear that this observation has been clarified in the intervening decades. Given the now extensive evidence that LPL plays critical functions in macrophage, neutrophil, eosinophil, and lymphocyte biology, further investigation of a role for LPL in infection and immunity is well warranted.

## Figures and Tables

**Table 1:** The kinases that regulate phosphorylation of LPL in response to various cell stimuli are listed, along with the inhibitor used to test the participation of each kinase. The kinases that phosphorylate LPL in other cell types, such as T cells and macrophages, have not yet been defined. References are numbered according to appearance in the text.

Cell Type	Receptor	Ligand	Inhibitor	Kinase inhibited	Ref.
Neutrophils	Fc*γ*R	IC	H89 Wortmannin	PKA PI3K	[49]

Neutrophils	FPR (high affinity)	fMLP	Ro-31-8220	PKC	[39]

Neutrophils	FPR-L1 (low affinity)	fMLP	LY294002 butanol Ro-31-8220	PI3K PLD PKC	[39]

Eosinophils	GMR	GM-CSF	siRNA	PKC*β*II	[60]

Abbreviations: Fc*γ*R: receptor for the Fc portion of immunoglobulin; IC: immune complexes; fMLP: N-formyl-L-methionyl-L-leucyl-L-phenylalanine; PKA: protein kinase A; PI3K: phosphatidylinositol 3-kinase; PKC: protein kinase C; FPR: formyl peptide receptor; FPR-L1: formyl peptide receptor-like 1; PLD: phospholipase D; GM-CSF: granulocyte macrophage colony stimulating factor; GMR: GM-CSF receptor.

## Abbreviations

LPL: L-plastin
ABD: Actin-binding domains
FRAP: Fluorescence recovery after photobleaching
Iba1: Ionized calcium binding adaptor molecule 1
BPB: Bromophenacyl bromide
BSA: Bovine serum albumin
FPR-L1: Formyl peptide receptor-like 1
APC: Antigen-presenting cell
SMAC: Supramolecular activation complex
IS: Immunological synapse.
